# Prediction of Plasticizer Property Based on an Improved Genetic Algorithm

**DOI:** 10.3390/polym14204284

**Published:** 2022-10-12

**Authors:** Yuyin Zhang, Ningjie Deng, Shiding Zhang, Pingping Liu, Changjing Chen, Ziheng Cui, Biqiang Chen, Tianwei Tan

**Affiliations:** National Energy R&D Center for Biorefinery, Beijing University of Chemical Technology, Beijing 100029, China

**Keywords:** plasticizer, substitution factor, machine learning, genetic algorithm, grid search algorithm

## Abstract

Different plasticizers have obvious differences in plasticizing properties. As one of the important indicators for evaluating plasticization performance, the substitution factor (SF) has great significance for product cost accounting. In this research, a genetic algorithm with “variable mutation probability” was developed to screen the key molecular descriptors of plasticizers that are highly correlated with the SF, and a SF prediction model was established based on these filtered molecular descriptors. The results show that the improved genetic algorithm greatly improved the prediction accuracy in different regression models. The coefficient of determination (*R*^2^) for the test set and the cross-validation both reached 0.92, which is at least 0.15 higher than the *R*^2^ of the unimproved genetic algorithm. From the results of the selected descriptors, most of the descriptors focused on describing the branching of the molecule, which is consistent with the view that the branching chain plays an important role in the plasticization process. As the first study to establish the relationship between plasticizer SF and plasticizer molecular structure, this work provides a basis for subsequent plasticizer performance and evaluation system modeling.

## 1. Introduction

A plasticizer is a substance or material that can increase the flexibility, processability, or expansion of plastics by reducing the glass transition temperature (Tg) [1,2,3]. In addition, many other properties of plastics are also affected by plasticizers, such as crystallization, melting and gel temperature, interaction with water, fire resistance, gas permeability, degradation rate, etc. [4]. As the most common plastic additive, worldwide production of plasticizers was around 6.4 million tons per year during the last decade. The global plasticizer market was valued at US$93.76 billion in 2019, and will reach US$111.38 billion by 2023 [5,6]. As the most important category of PVC plasticizers, phthalates are the most used plasticizers in the world. However, the use of phthalates has caused concern and controversy due to the migration phenomenon toward elements in contact with them (medical and childcare articles) and bioaccumulation in the environment [7,8]. Therefore, the current goal is to still find new plasticizers to satisfy the numerous applications of plastic products. The most traditional method for discovering new plasticizers is experimental exploration, which includes structure synthesis, property analysis, and measurement. Although experimental exploration is intuitive and accurate, it has been carried out in an inefficient manner for a long time. In addition, experimental exploration has high requirements on equipment, experimental environment, and the professional knowledge of researchers. It has limitations of being greatly affected by the external environment, long cycle, and high cost, which make it difficult to accelerate the development of plasticizers. Therefore, it is necessary to develop more effective plasticizers research methods to shorten the research cycle.

In the past decade, machine learning (ML) has become a powerful tool for accelerating material development. ML publishing activities for chemicals and materials are growing exponentially [9,10,11]. In particular, the publication of some polymer databases has laid a data foundation for ML-related research on polymer materials. A representative database is NanoMine [12], which builds an extensible data representation for data on the material composition, properties, and microstructure of a polymer nanocomposite. Another similar database that can be used for polymer material design is PoLyInfo, which collects information such as polymer name, chemical structure, sample processing method, measurement conditions, properties, monomers used, and polymerization method [13]. ML method can extract knowledge from existing data, gain insights, and produce reliable results, especially for high-dimensional data classification and regression. Therefore, the method of ML can help during the development and research of materials. Especially in polymer-related fields, the emerging of polymer informatics aims to provide tools to accelerate performance prediction (and design) through alternative ML methods based on reliable data [14]. Stephen Wu et al. gave a systematic review of the potential and challenges of the latest polymer informatics [15]. Some recent research on polymer ML has focused on performance indicators such as Tg and atomization temperature [16]. Chiho Kim et al. established a polymer informatics platform, which uses ML methods to link the key characteristics and performance of polymers, and can predict various important polymer properties on demand [16]. Ghanshyam Pilania et al. established the Tg prediction model of polyhydroxyalkanoate (PHA) homopolymers and copolymers based on the ML method [17]. Similar work has also been performed by Yun Zhang et al., which uses the Gaussian process regression model to establish a Tg prediction model of polymer [18]. In terms of data set sensitivity, Anurag Jha et al. explored the impact of data set uncertainty on the prediction of polymer Tg by ML methods [19]. In the prediction of polymer functions other than physical properties, a typical machine learning application is the prediction of the performance of polymer filtration membranes (polyvinylidene fluoride, polyethersulfone, and polysulfone filtration membranes) [20]. Wang et al. presented a novel deep learning approach that combined convolutional neural networks with multi-task learning for building quantitative correlations between microstructures and property values of nanostructured polymers [21]. However, as an important aid for adjusting the properties of polymers, the application of ML in the field of plasticizer performance evaluation and prediction has not been reported as far as we know.

Different plasticizers produce different plasticizing effects due to the strength of the plasticizer-polymer and the plasticizer-plasticizer interaction [22]. Plasticizers generally contain two structural components: polar and non-polar parts. The polar part of the molecule must be able to reversibly bind to the polymer to soften the polymer, while the non-polar part allows controlling the interaction between the polymers [1]. Chandola et al. proposed a more accurate plasticization interpretation model, which established a relationship between performance (specific volume, viscosity, etc.) and variables (molecular weight, terminal group content, etc.), allowing it to predict the behavior of 25 PVC plasticizers [23]. In addition to the physical property parameters, like Tg and atomic atomization temperature, etc., an important index to measure the comprehensive performance of plasticizers is the “Substitution Factor” (SF) [22,24,25,26]. The definition of SF is the amount of another plasticizer, other than one specific plasticizer (such as DOP), in order to plasticize equivalently, according to the following formulae:

(1) $$\mathrm{Substitution}\;\mathrm{Factor}\;\left(\mathrm{SF}\right)=\left(\frac{\mathrm{PHR}\;\mathrm{plasticizer}\;\mathrm{at}\;\mathrm{Durometer}\;80}{\mathrm{PHR}\;\mathrm{DOP}\;\mathrm{at}\;\mathrm{Durometer}\;80}\right)$$

where PHR is per 100 parts of polymer, which represents the parts by weight of plasticizer per 100 parts of resin required to produce a plasticized PVC resin of a particular hardness on a certain Durometer scale [27]. It was found that the SF is consistent over the plasticizer level range from 20 to 90 phr, and the value of SF usually increased as the molecular weight of the plasticizer increased [1,28]. Substitution factors of a large number of commercial plasticizers have been evaluated in order to evaluate and adjust the properties and performance of additives, with DOP always chosen as a reference [29]. In addition, the SF of the plasticizer also had great significance for the cost estimation of obtaining a specific hardness product [30]. Therefore, obtaining the SF of plasticizers accurately and effectively provides important support not only for evaluating the properties of plasticizers, but also for evaluating the economic feasibility of plasticizers.

Obtaining the SF of plasticizer through experiments has a long research period and high cost, which brings disadvantages in a large number of potential plasticizer screenings. On the other hand, the existing plasticizer substitution factor data has not been fully utilized. Considering that the main types of plasticizers are esters, the similar methods and experience of known esters quantitative structure-property relationship (QSPR) is used to build a predictive evaluation model between SF and plasticizer molecules [31,32]. In this work, traditional and improved genetic algorithms (GA), as well as grid search algorithms, were used in combination with ML methods, such as support vector machines (SVM), random forests (RF), and partial least squares (PLS), to screen important molecular descriptors of plasticizer molecules, and to model the difference between SF and molecular descriptors. The results showed that the support vector machine model constructed by screening the descriptors with an improved genetic algorithm and further dimensionality reduction by principal component analysis (PCA) showed good prediction results. A combination of the grid search algorithm and SVM also showed good prediction results, although were weaker than the optimal model. The screened descriptors were also analyzed and the molecular features related to plasticizer substitution factors were interpreted, providing theoretical support for the design of new plasticizer molecules outlined in the next section.

## 2. Methods

The calculations used in this article were implemented in Python 3.7 by either calling the toolbox or writing scripts with internal functions.

### 2.1. Data Set

The original SF data came from the PVC Handbook [1], and the molecular structures from PubChem database [33]. The SF data of 26 modules are provided. The original data set can be found in Table 1, and the structures of all these plasticizers are listed in Table S1.

The number of plasticizers included in the dataset in this study was limited, though we used almost all the SF data in the PVC Handbook under the premise of ensuring a uniform data source. The reason for this is that the number of plasticizers available for industrial applications is very limited. This directly hindered the acquisition of the SF, which is an economic evaluation index. The relevant publicly available database was not reported either. To ensure the reliability of the data, the uniformity of molecular structure sampling was checked, and the descriptor entries for individual molecules were calculated (over 1200) to the maximum extent to increase the richness of the data in the lateral dimension.

Although polyethylene glycol and glycerol are widely used to modify the properties of polymer-based electrolytes, and play a major role in conductivity enhancement and enhancing the flexibility of films, it is difficult to obtain accurate quantitative molecular descriptors due to their nature as polymers, and makes it impossible to establish a unified model with single-molecule plasticizers. Therefore, low molecular weight polymer plasticizers were temporarily excluded from this study.

The structural optimization of all molecules was performed in HyperChem 8.0.8 (Hypercube, Inc., Gainesville, FL 32601, USA) [34]. The semi-empirical AM1 method and the Fletcher-Reeves algorithm were adopted in the optimization process until the root mean square gradient was less than 0.01. For each molecule, 1664 molecular descriptors were calculated using E-dragon [35] (the electronic remote version of Dragon 5.4 on 28 March 2006) and 1277 descriptors were reserved after deleting all the constant values. The Kennard-Stone algorithm [36] was used to divide the original data set into a training set and a test set, 21 and 5 data, respectively. All models were evaluated by 5-fold cross-validation (5-fold CV) on the training set and evaluated on the test set.

### 2.2. Genetic Algorithm

**Figure 1:**
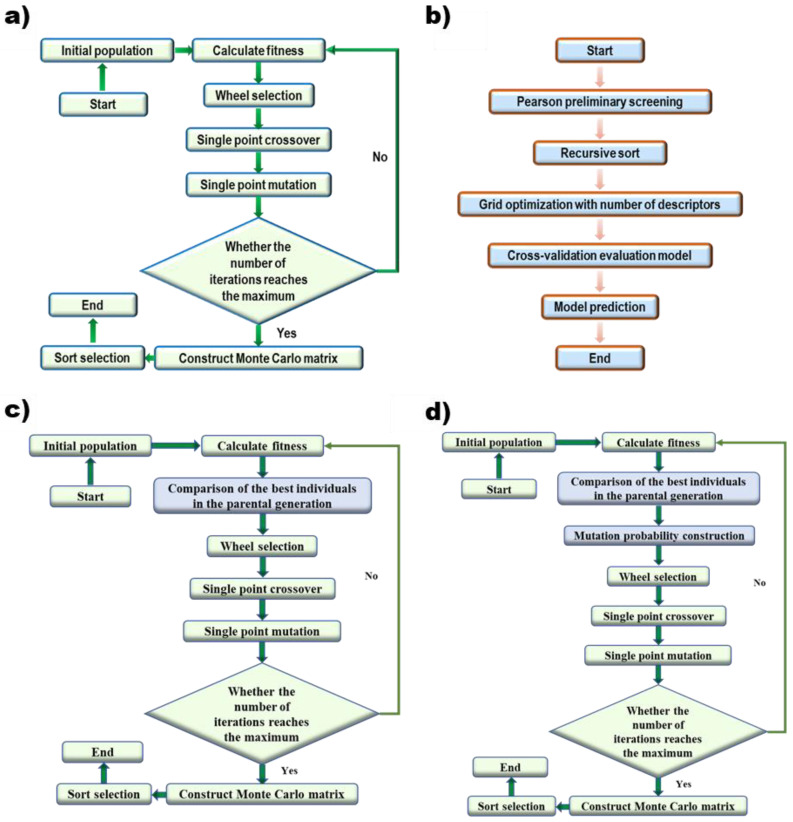
Algorithm flow of (**a**) Genetic Algorithm, (**b**) Grid Search, (**c**) GA with the elite retention method, and (**d**) GA with the mutation-selection strategy based on evolutionary history.

The selection of descriptors is directly related to the predictive ability of the model. Genetic algorithms [37,38] are widely used in the selection of descriptors. Genetic algorithm (Figure 1a) uses the objective function (fitness) to evaluate individuals and perform genetic operations of selective crossover mutation to obtain a new population. It keeps the important features in the parent generation while looking for better patterns in the iterative process. After all iterations are completed, a Monte Carlo sample set is established based on the optimal individuals of each generation, and all descriptors are statistically sorted to obtain the optimal descriptor set.

Each generation of population includes N individuals and each individual is a string consisting of 0 and 1, where 1 and 0 mean the corresponding descriptor is selected or not. The fitness was calculated using a 5-fold cross-validation according to Equation (2):

(2) $$\mathrm{Fitness}\;\left(x_{i}\right)=CV_{\left(5\right)}=\frac{1}{5}\overset{5}{\underset{i=1}{\sum }}R^{2}$$

where $x_{i}$ is the individual and $R^{2}$ represents the coefficient of determination for each round of cross-validation. The roulette wheel strategy is used to select the next generation of individuals, according to Equation (3):

(3) $$\{\begin{array}{c} NP_{i}=\overset{i}{\underset{j=1}{\sum }}p_{i}\\ NP_{0}=0 \end{array}$$

where *NP_i_* is cumulative probability and *P_i_* is the selection probability of each individual. *P_i_* can be calculated by:

(4) $$p_{i}=\frac{\mathrm{fitness}\;\left(x_{i}\right)}{\sum_{i=1}^{NP}\mathrm{fitness}\;\left(x_{i}\right)}$$

N times of Monte Carlo sampling are performed to construct the offspring population based on the cumulative probability of the parent population, where N is the number of individuals in each generation of the population. A random number *r* is generated between 0 and 1 for each sample, and individual *i* was selected if *NP_i_*_−1_ ≤ *r* ≤ *NP_i_.* The probability of crossover and mutation is *Pc* and *Pm*, respectively, and crossover or mutation of a single point is implemented if the random number *Rc*(*Rm*) < *Pc*(*Pm*). All the specific parameters are listed in Table 2.

In order to find the global optimal as far as possible, the genetic algorithm was optimized. First, the elite retention method was added to the original selection method, which means the best individual of the offspring will be replaced by the best individual of the parent if the best individual of the offspring was inferior to the best individual of the parent (Figure 1c). Second, a mutation-selection strategy based on evolutionary history was added to the algorithm (Figure 1d). For every single point, a new mutation probability $Pm_{i}$ is generated based on the counting of the gene encoding and fitness of the best individual in the last 50 iterations. The calculation of $Pm_{i}$ follows Equation (5):

(5) $$Pm_{i}=\{\begin{array}{cc} Min-Max\;Normalization\left(\frac{\sum_{i=1}^{50}a_{i}b_{ij}}{\sum_{i=1}^{50}b_{ij}}\right), & \overset{50}{\underset{j=1}{\sum }}b_{ij}\neq 0\\ 0.5, & \overset{50}{\underset{j=1}{\sum }}b_{ij}=0 \end{array}$$

where *a_i_* represents the fitness of the *i*th best individual, and *b_ij_* represents the value state (0 or 1) of the *j*th descriptor of the *i*th best individual.

### 2.3. Grid Search

The Grid Search algorithm (Figure 1b) is an algorithm to find the optimal parameters of the kernel function. The raw data is normalized and the Pearson coefficient between arbitrary descriptors is calculated before grid optimization. A total of 16 descriptors, whose correlations between descriptors were not more than 0.5 and the correlations between descriptors and predicted properties were not lower than 0.1, were screened out. The obtained descriptors and the number of selected descriptors were used as a hyperparameter for grid search, and an early-stop strategy was used to prevent overfitting [39]. The evaluation of the final model was performed with 5-fold cross-validation on the training set. All the specific parameters are listed in Table 3.

### 2.4. Regression Algorithm

PLS is one of the most widely used algorithms at present. It can acquire the mutually orthogonal eigenvectors of the independent variables and the dependent variables by projecting the high-dimensional data space of the independent variable and the dependent variable to the low-dimensional space, and then establish the univariate linear regression relationship between the eigenvectors of the independent variable and the dependent variable. Not only can it overcome the problem of collinearity, it emphasizes the interpretation and prediction effect of the independent variable on the dependent variable when selecting the feature vector, removes the influence of the regression unhelpful noise, and causes the model to contain the least number of variables.

Random Forest is a supervised ensemble learning algorithm [40]. Through the bootstrap resampling technology, the algorithm randomly selects k samples from the original training sample set with replacement to generate a new training sample set, and then generates a plurality of classification or regression trees to form a forest based on these self-service sample sets. The classification or regression result of the new data is determined by the score formed by the votes of each tree.

Support Vector Machine (SVM) is a machine learning algorithm [41], which separates data by projecting the data into a high-dimensional space to construct a hyperplane [42], proposed on the basis of statistical theory. The SVM algorithm has many advantages, such as being able to solve both classification problems and numerical prediction problems, not be seriously affected by noise data, and not prone to over-fitting. The main disadvantage is that the result of SVM is difficult or impossible to explain.

The coefficient of determination [43] ($R^{2}$) was employed in this study to evaluate the results of cross-validation and prediction. $R^{2}$ is calculated according to Equation (6):

(6) $$R^{2}=1-\frac{\sum_{i=1}^{n}\left(y_{pred,i}-y_{exp,i}\right)}{\sum_{i=1}^{n}\left(y_{pred,i}-{\bar{y}}_{exp,i}\right)}$$

where $y_{exp,i}$ and $y_{pred,i}$ are the actual and predicted values, *i* is the data record number, ${\bar{y}}_{exp,i}$ is average of the actual values, and *n* is total number of data. The model that gives a higher $R^{2}$ means that model can give a more accurate prediction.

## 3. Results and Discussion

### 3.1. Data Set Division and Feature Screening

**Figure 2:**
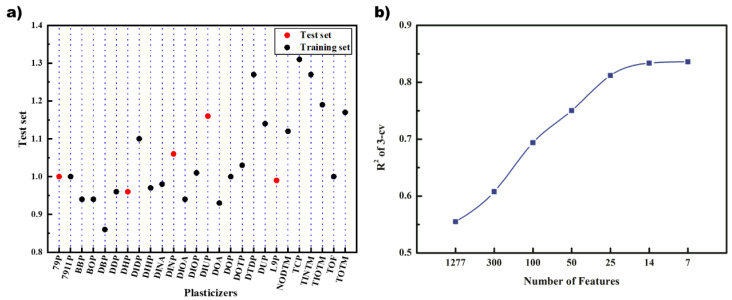
(**a**) Data set division; (**b**) convergence of genetic algorithm iteration.

Kennard-Stone algorithm divided 26 data into training set and test set, which contained 21 and 5 data, respectively. The result is shown in Figure 2a, where the red point represents the test set, and the blue point represents the training set. It can be seen that the substitution factors are evenly distributed in the entire range, and the range of the training set can cover the data of the test set.

The 1277 molecular feature descriptors that passed the preliminary screening were screened by GA and Grid Search, respectively. First, GA and three regression methods were used to screen the descriptors, respectively. The results of GA-PLS, which present the best results, are shown in Figure 2b. It can be seen that the *R*^2^ of cross-validation on the training set gradually increased as the descriptors decreased. Finally, the *R*^2^ stabilized at about 0.8 when the number of descriptors reached 14 and 7.

### 3.2. Grid Search

**Figure 3:**
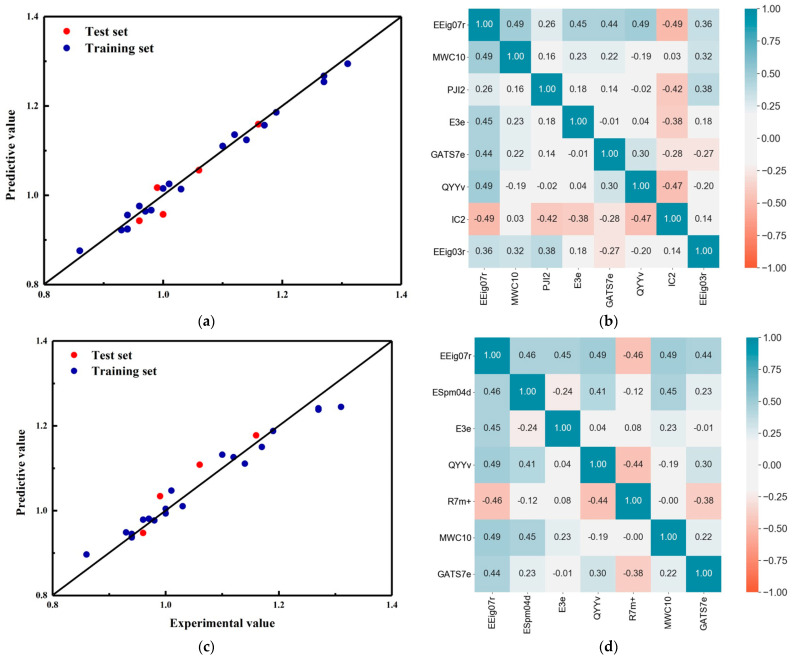
The prediction results of different regression models based on grid optimization and the correlation of descriptors. (**a**) And (**b**) SVM; (**c**) and (**d**) RF.

The models were constructed under different numbers of descriptors (Tables S2 and S3). The cross-validation results for the Grid + RFR model fluctuated between 0.65 and 0.75 as the number of descriptors increased, with a maximum of seven descriptors in the test set. The cross-validation and test set results for the grid + SVM model generally increased and then decreased as the number of descriptors increased, reaching a maximum of 10 for the cross-validation and 8 for the test set. Combining the cross-validation and test set results, the Grid + RFR model ultimately selected a total of seven descriptors: EEig07r, ESpm04d, E3e, QYYv, R7m+, MWC10, and GATS7e. The Grid + SVM model ultimately selected EEig07r, MWC10, PJI2, E3e, GATS7e, QYYv, IC2, and EEig03r for a total of eight variables. The correlation between the prediction results and descriptors of the different regression models based on the Grid Search is shown in Figure 3.

In terms of prediction results, both models showed excellent prediction performance. The data from both models on the training and test sets were evenly distributed around the standard line with very little deviation. In terms of descriptor correlation, the correlation of descriptors in both models was satisfactory. The reason for this is that the Grid Search selected descriptors were directly based on the Pearson correlation coefficient between the descriptors. Therefore, whatever combination of descriptors the grid search chose, it had good linear irrelevance.

### 3.3. Genetic Algorithm

#### 3.3.1. GA Modelling Approach

The *R*^2^ of the different numbers of descriptors selected by the GA were sorted in to the training set, cross-validation, and test set (Tables S4 and S5). The *R*^2^ of cross-validation of GA + PLS model increased with the rise of the descriptors number. However, on the test set, *R*^2^ first increased and then decreased with the increase of the number of features, and reached the maximum at 9 features. The results of GA + RF and GA + SVR on cross-validation both first increased and then decreased with the increase of the number of descriptors, reaching the maximum at 4 and 10, respectively, and showed a trend of decreasing fluctuation on the test set (Tables S6 and S7).

**Figure 4:**
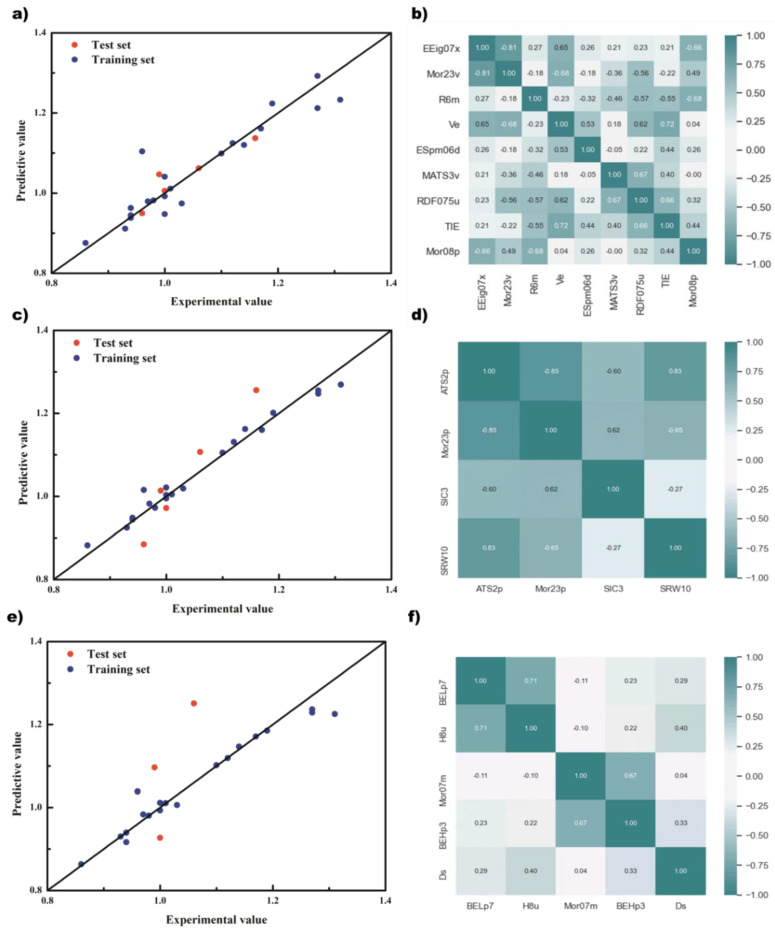
The prediction results of different regression models based on genetic algorithm and the correlation of descriptors. (**a**) And (**b**) PLS; (**c**) and (**d**) RF; (**e**) and (**f**) SVM.

Considering the results of cross-validation and the test set, GA+PLS finally selected 9 descriptors, namely EEig07x, Mor23v, R6m, Ve, ESpm06d, MATS3v, RDF075u, TIE, and Mor08p; GA + RFR selected 4 descriptors, namely ATS2p, Mor23p, SIC3, and SRW10; GA + SVR selected 5 variables, namely BELp7, H8u, Mor07m, BEHp3, and DS. The prediction results of different regression models based on GA and the correlation of descriptors are shown in Figure 4.

From the prediction results of the model, the points of the GA + PLS and GA + RF models were relatively evenly distributed on both sides of the standard line and relatively concentrated, and there was less obvious deviation from the standard line. However, there was overfitting in the GA + SVM model. The data on the training set were very consistent with the standard line, but the data on the test set had a far deviation, and the coefficient of determination was only 0.27. Although there was only one obvious deviation point, this made the method unconvincing in terms of prediction accuracy. Considering both the prediction results and descriptor correlation, the GA + PLS model achieved the best results. It can be seen that when the training set was relatively consistent, the points of the test set also had a small deviation, and the coefficient of determination was also 0.85. In addition, any two descriptors of the GA + PLS model were basically linearly independent, as shown in Figure 4b, which avoided the negative effects of excessive high-dimensionality on the model, and also showed that the model is established reasonably. In contrast, the descriptor correlation of GA + RF was much worse, although the prediction results were good.

#### 3.3.2. GA Algorithm Improvements

**Figure 5:**
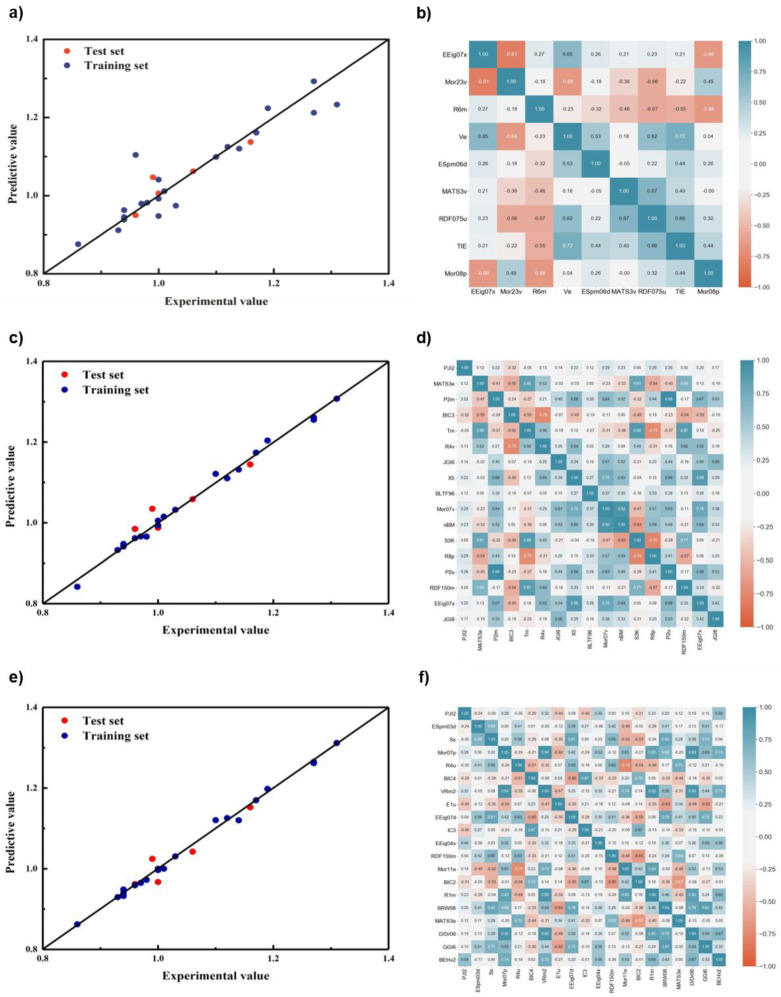
The prediction results of different regression models based on genetic algorithm and the correlation of descriptors. (**a**) And (**b**) GA-O; (**c**) and (**d**) GA-RE; (**e**) and (**f**) GA-REC.

After reconstructing the algorithm structure of the improved GA, two modeling approaches, PLS and SVR, were performed when the descriptors were filtered to 25 and 14, respectively, and it was found that the results of both modeling approaches were better when the descriptors were 25 than when the descriptors were 14. The results of modeling approach PLS were better than SVR approach in general. The PLS results are shown in Figure 5. Here, the original GA, GA with elite retention, and GA with both variation probability and elite retention are referred to as GA-O, GA-RE, and GA-REC, respectively, and the results of the models showed that, compared with GA-O, GA-RE had a more concentrated distribution of points on both sides of the standard line in the training set, and its *R*^2^ improved from 0.8754 to 0.9933. In the test set, GA-RE had no individual points with large deviation, and its *R*^2^ increased from 0.8453 to 0.8805. There is almost no difference between the distribution of GA-RE and GA-REC in the training set, but GA-REC had a more reasonable distribution of points on both sides of the standard line, and its *R*^2^ increased from 0.8805 to 0.8953.

From the correlation results of descriptors, GA-RE finally chose PJI2, MATS3e, P2m, BIC3, Tm, R4v, JGI6, X5, BLTF96, Mor07v, nBM, S3K, R8p, P2u, RDF150m, EEig07x, and JGI8. GA 3.0 chose PJI2 ESpm03d, Ss, Mor07p, R4u, BIC4, VRm2, E1u, EEig07d, IC3, EEig04x, RDF150m, Mor11e, BIC2, R1m, SRW08, MATS3e, D/Dr06, GGI6, and BEHv2. Although the number of descriptors selected for modeling, as the GA improved, increased, rising from 9 to 17 to 20, the correlation of descriptors decreased instead in proportions greater than 0.5, from 0.36 to 0.30 to 0.27, indicating that the model was constructed to cover more molecular information.

#### 3.3.3. Combination of GA and PCA

**Figure 6:**
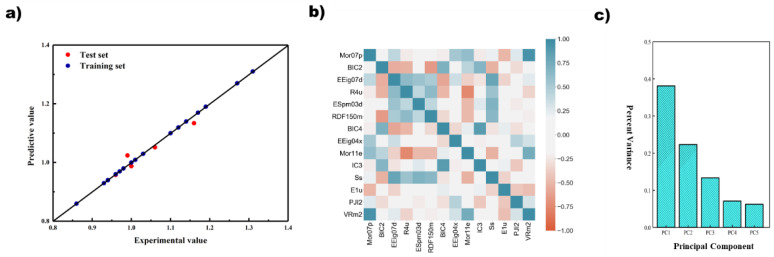
Results of the regression model based on GA-REC + PCA + SVR. (**a**) Prediction results; (**b**) correlation of descriptors; (**c**) principal component results.

To further compress the variables used at input, further dimensionality reduction using principal component analysis (PCA) was attempted for the filtered 25 and 14 descriptors in model construction for PLS and SVR. The results are shown in Table S8. From the table, it can be seen that the overall reduction of each model is much lower compared to the results without PCA. However, an optimal result was found in which the 14 descriptors were reduced to five principal components using PCA, and the *R*^2^ on the cross-validation and test sets were 0.9182 and 0.9181, respectively, which achieved better results compared to the previous ones. Additionally, the five principal components obtained at the end contained 87% of the principal component information. The results are shown in Figure 6.

Table 4 shows the results of comparing several models for the same number of five descriptor variables. The PCA + SVR approach with the same GA is better than the PLS approach. For both methods, it is clearly observed that the improved GA-REC algorithm is much better than the GA-RE algorithm. There is a significant improvement in the training set, cross-validation, and test set on PLS, and an improvement in the cross-validation values on PCA + SVR while retaining the excellent results on the training and test sets. Thus, it can be concluded that the improved GA-REC algorithm structure was more suitable for the goal of finding global variables.

### 3.4. Feature Analysis

This description is based on the analysis of the best performing GA-REC + PCA + SVR model. Among the 14 features for principal component analysis (Table 5), there are mainly GETAWAY, WHIM, Edge adjacency indices, Constitutional descriptors, topology, 3D-MoRSE descriptor, information index, and RDF descriptor index. Among them, GETAWAY and WHIM mainly encode information related to screenshots and local bases, and use them at the same time to increase modeling capabilities. The edge adjacency index is based on the two-dimensional topological structure of molecular processing, and can establish molecular systems containing rings, continuous bonds, and heteroatoms. The environment can prove the characteristics of atom-breaking electrons and atoms in molecules, describing a certain atom or molecular piece, and not encoding relevant overall topology and geometric structure information. The 3D-MoRSE descriptor is the basic resource obtained by the leaky basis function centered on the distance between atoms (0.5A to 15.5A) obtained from the highest numerical longitude of quantum chemistry.

A summary of these descriptors and their meanings is presented in Table 6. As can be seen, the descriptors used for modeling focus on describing the structure of the molecule, especially in relation to the molecular branching. This is not only related to the important role of branching in plasticizer plasticization, but also to the fact that this data set is mainly related to the benzene ring as the central structure of the molecule. Thus, the known results can consider that certain properties of molecular branching have a greater weight in the prediction of plasticizer substitution factors. This also provides a basis for future principle interpretation and model exploration of plasticizers.

### 3.5. Prediction of Several Potential Bio-Based Plasticizers

With the development and utilization of biomass energy sources, the research on biomass plasticizers has been increasing. Therefore, the model constructed in this work was applied to predict the SF of several biobased plasticizers in order to provide some exploratory directions for the development of biobased plasticizers. See Table S9 in the attached table for details. Among them, the SF of Dimethyl furan-2,5-dicarboxylate and isosorbide dioctanoate were compared with available experimental values [44] and were in general agreement. Moreover, the predicted value of SF for tetrakis(butyl)benzene-1,2,4,5-tetracarboxylate, which is an environmentally friendly plasticizer and more synthetic, was only 1.03.

## 4. Conclusions

As an important index to measure the performance and economy of plasticizers, the substitution factor has been attracting attention from the industry. However, research and model building on the relationship between SF and plasticizer molecular structures, especially model building based on machine learning algorithms, have not been reported. Based on the reported SF data of plasticizers, this work used a genetic algorithm and grid search algorithm to screen the molecular descriptors of different plasticizer molecules and establish the model between key descriptors and SF. A genetic algorithm with “variable mutation probability” (GA-REC) was also developed in this work to screen the key molecular descriptors of plasticizers that were highly correlated with the SF, and a SF prediction model was then established based on these filtered molecular descriptors. The combined results indicate that the GA-REC + PCA + SVR model will be more suitable for this system. Its *R*^2^ on the test set reached 0.9181, with perfect fit on the training set, and 0.9192 with cross-validation results, indicating that the model had good generalization ability. The improved genetic algorithm has greatly improved the prediction accuracy in different regression models. The coefficient of determination (*R*^2^) for the test set and the cross-validation was at least 0.15 higher than the *R*^2^ of the unimproved genetic algorithm. The filtered descriptors also covered relatively complete molecular information, such as rings, heteroatoms, local branches, etc., which also illustrated the scientific validity of the model from the side. The descriptors also revealed the importance of molecular branching features in the action of plasticizers. This conclusion is consistent with the basic judgment of the plasticizer principle. The model constructed in this work was also applied to predict the SF of several biobased plasticizers. Among them, the SF of Dimethyl furan-2,5-dicarboxylate, and isosorbide dioctanoate were compared with the available experimental values and were in general agreement. As the first study to establish the relationship between plasticizer SF and plasticizer molecular structure, this work compared the effectiveness of major machine learning approaches adapted to low data volumes, and provides a basis for subsequent modeling of plasticizer performance and evaluation systems.

## Figures and Tables

**Table 1:** Plasticizer Acronyms, Chemical Compositions, and Substitution Factors.

Acronym		Chemical Structure	Subst. Factor
Phthalates	BBP	butyl,benzyl	0.94
	BOP	butyl,2-ethylhexyl	0.94
	DHP	di(isohexyl)	0.96
	DIHP	di(isoheptyl)	0.97
	DOP	di(2-ethylhexyl)	1
	DIOP	di(isooctyl)	1.01
	DINP	di(isononyl)	1.06
	DIDP	di(isodecyl)	1.1
	DIUP	di(isoundecyl)	1.16
	DTDP	di(isotridecyl)	1.27
Linear phthalates	DBP	di(n-butyl)	0.86
	79P	di(linearC7,C9)	1
	L9P	di(linearnonyl)	0.99
	7911P	di(linearC7,C9,C11)	1
	DUP	di(linearC11)	1.14
Trimellitates	NODTM	tri(n-C8,C10)	1.12
	TOTM	tri(2-ethylhexyl)	1.17
	TIOTM	tri(isooctyl)	1.19
	TINTM	tri(isononyl)	1.27
Adipates	DOA	di(2-ethylhexyl)	0.93
	DIOA	di(isooctyl)	0.94
	DINA	di(isononyl)	0.98
Phosphates	DDP	isodecyl,diphenyl	0.96
	TOF	tri(2-ethylhexyl)	1
	TCP	tricresyl	1.31
Others	DOTP	di(2-ethylhexyl)terephthalate	1.03

**Table 2:** Parameters of Genetic algorithm.

Parameter	Value
Number of evaluations	200
Fitness	*R*^2^CV in 5-fold cross-validation
Number of chromosomes	50
Probability of mutation	0.01
Probability of cross-over	0.5

**Table 3:** Parameters of Grid Search.

Parameter	Value
The multiplicity of cross-validation in grid optimization	5
Number of repetitions of grid optimization	10
The minimum mean_test_score change rate that triggers early_stop	0.01
Lower bound of descriptor and activity correlation in Pearson correlation	0.1
Descriptor pairwise correlation upper bound in Pearson correlation	0.5
Compression range of characteristic data	(0.1)
Multiplicity of cross-validation of fitted model	5

**Table 4:** *R*^2^ of different models on training set, cross-validation, and test set.

Method		n-Feature	tr_r2	cv_r2	te_r2
GA-RE	PLS	5	0.8071	0.5088	0.5353
	PCA + SVR	5	1	0.7634	0.9201
GA-REC	PLS	5	0.8698	0.7211	0.8862
	PCA + SVR	5	1	0.9182	0.9181

**Table 5:** Description of 14 descriptors in GA-REC+PCA.

Descriptor	Description	Class
PJI2	2D Petitjean shape index	Topological indices
ESpm03d	Spectral moment 03 from edge adj. matrix weighted by dipole moments	Edge adjacency indices
Ss	sum of Kier-Hall electroTopological states	Constitutional descriptors
Mor07p	signal 07/weighted by polarizability	3D-MoRSE descriptors
R4u	R autocorrelation of lag 4/unweighted	GETAWAY descriptors
BIC4	Bond Information Content index (neighborhood symmetry of 4-order)	Information indices
VRm2	average Randic-type eigenvector-based index from mass weighted distance matrix	Eigenvalue-based indices
E1u	1st component accessibility directional WHIM index/unweighted	WHIM descriptors
EEig07d	Eigenvalue 07 from edge adj. matrix weighted by dipole moments	Edge adjacency indices
IC3	information content index (neighborhood symmetry of 3-order)	Information indices
EEig04x	Eigenvalue 04 from edge adj. matrix weighted by edge degrees	Edge adjacency indices
RDF150m	Radial Distribution Function—150/weighted by mass	RDF descriptors
Mor11e	signal 11/weighted by Sanderson electronegativity	3D-MoRSE descriptors
BIC2	Bond Information Content index (neighborhood symmetry of 2-order)	Information indices

**Table 6:** Structure and predicted values of several bio-based plasticizers.

No.	Structure	SF Predicted Value	SF Experimental Value
1	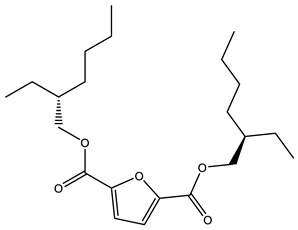	1.16	1.05
2	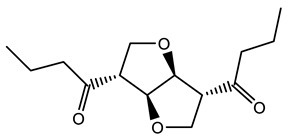	0.91	
3	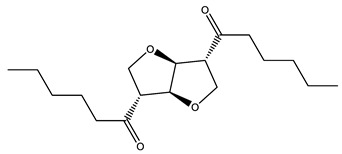	0.98	
4	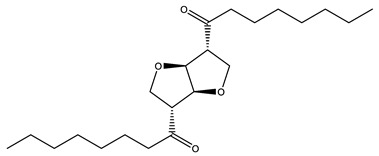	1.04	1.03
5	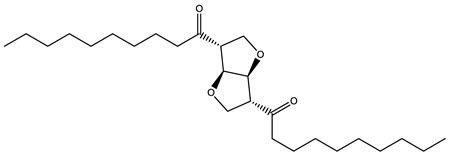	1.09	
6	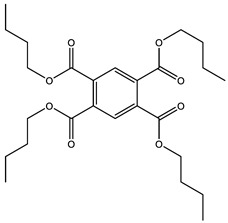	1.03	

## Data Availability

The datasets supporting the conclusions of this article are included within the article and its Supplementary Materials.

## Supplementary Materials

The following supporting information can be downloaded at: https://www.mdpi.com/article/10.3390/polym14204284/s1, Table S1: Molecular Structure; Table S2: Grid-SVR results under different numbers of descriptors; Table S3: Grid-RFR results under different numbers of descriptors; Table S4: GA-PLS Top 12 when molecular descriptors are screened to 25; Table S5: GA-PLS Top 12 when molecular descriptors are screened to 13; Table S6: GA-SVR results under different numbers of descriptors; Table S7: GA-RFR results under different numbers of descriptors; Table S8: *R*^2^ for different models on training set, cross-validation and test set; Table S9: SF projections for some potential plasticisers.
