# Supplementary material for: Prediction of Plasticizer Property Based on an Improved Genetic Algorithm
Source: Polymers (Basel). 2022 Oct 12;14(20):4284. doi: 10.3390/polym14204284 (PMC9607559; doi:10.3390/polym14204284)
Supplement: Supplementary file 1 [file polymers-14-04284-s001.zip › polymers-1940422-supplementary.pdf]

polymers

Supplementary Materials

Prediction of Plasticizer Property Based on an Improved Genetic Algorithm

Yuyin Zhang, Ningjie Deng, Shiding Zhang, Pingping Liu, Changjing Chen, Ziheng Cui, Biqiang Chen and Tianwei Tan

Table S1. Molecular Structure.

No.	Name	Structure
1	TOTM	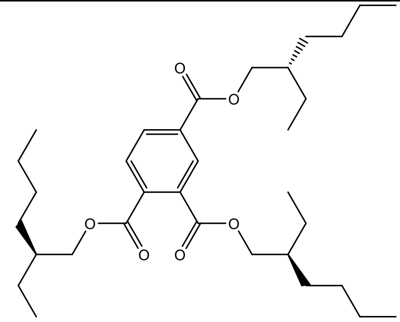
2	TOF	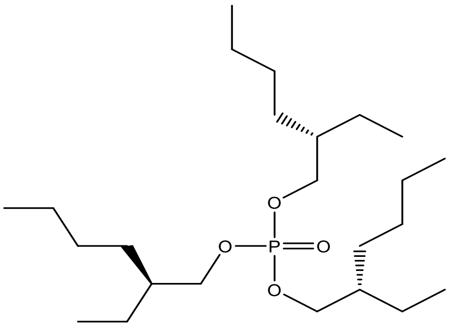
3	TIOTM	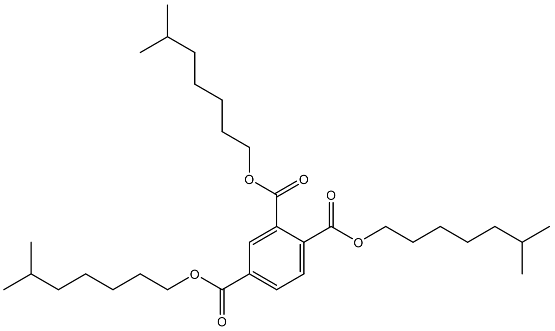
4	TINTM	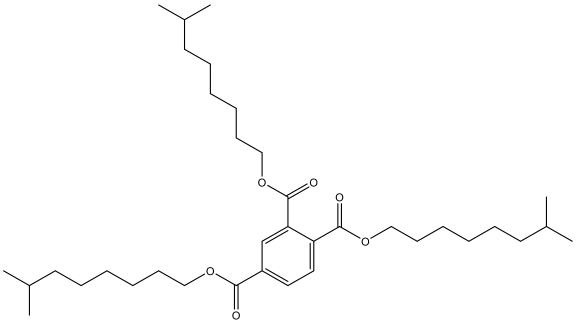

5	TCP	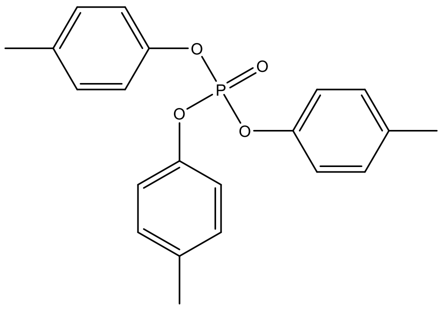
6	NODTM	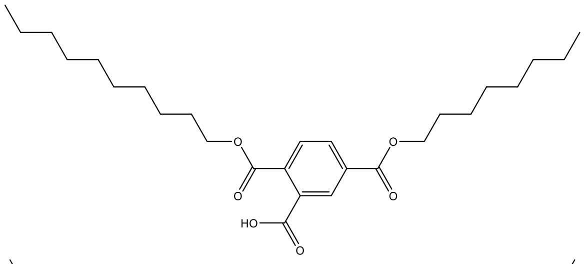
7	L9P	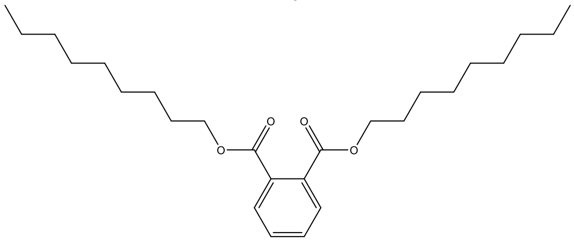
8	DUP	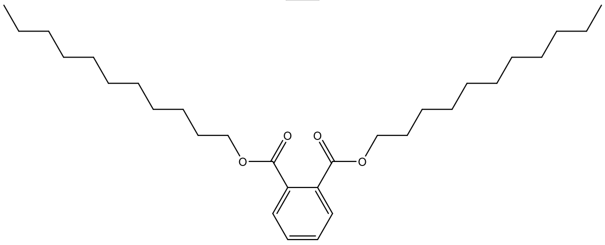
9	DIDP	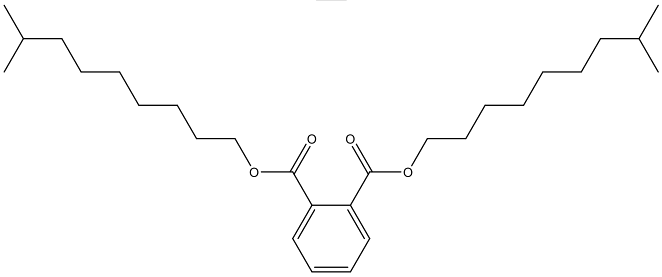
10	DOTP	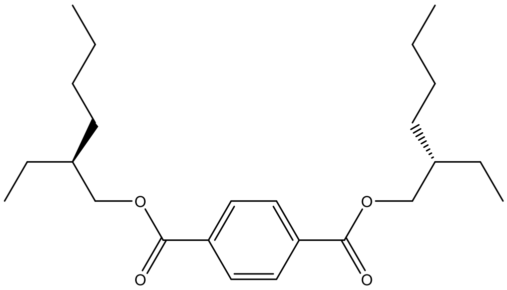

11	DOP	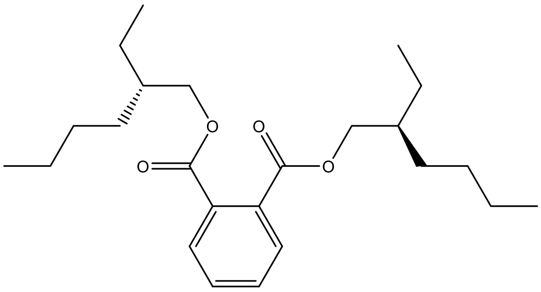
12	DOA	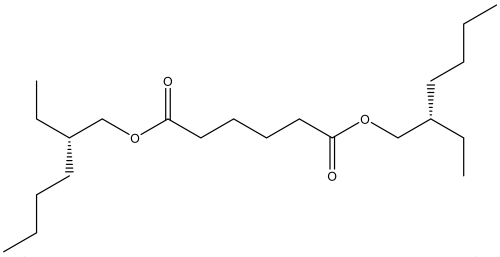
13	DIUP	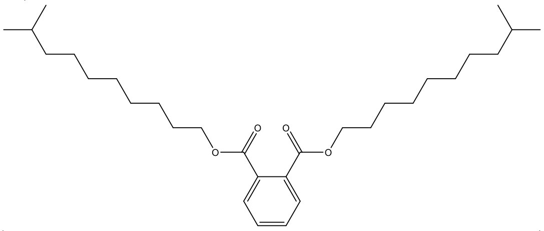
14	DIOP	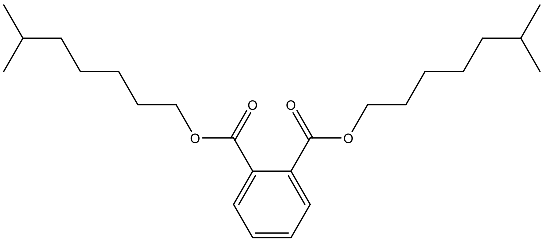
15	DIOA	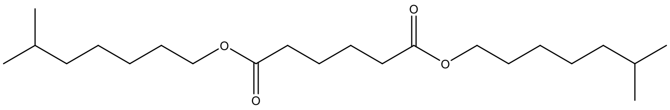
16	DINP	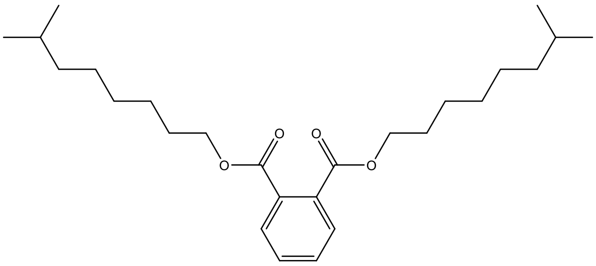
17	DINA	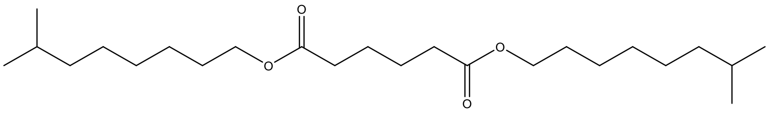
18	DIHP	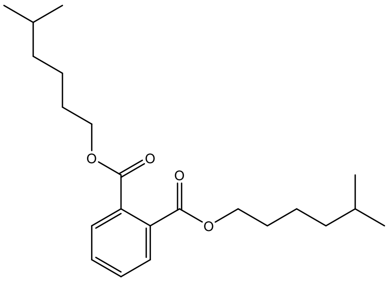

19	DIDP	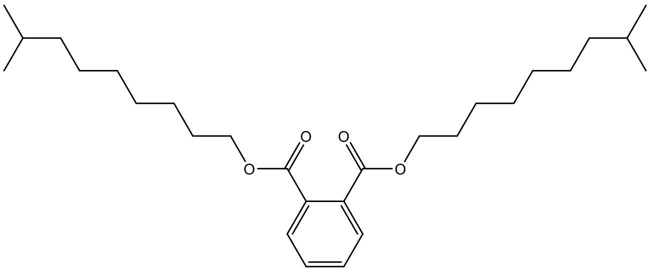
20	DHP	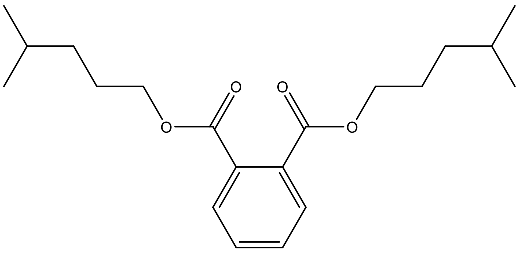
21	DDP	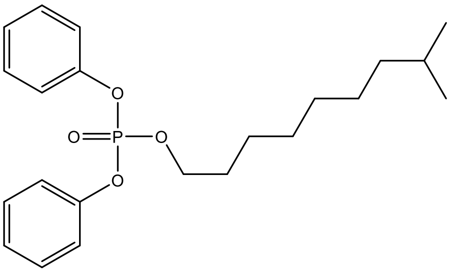
22	DBP	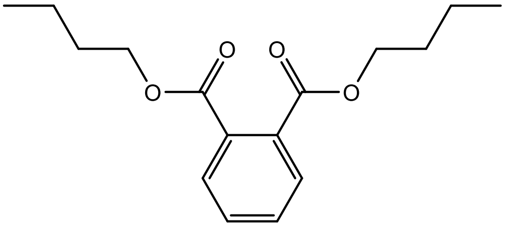
23	BOP	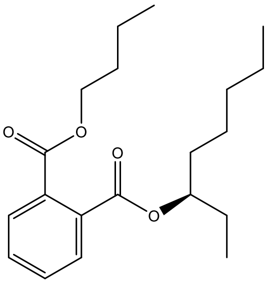
24	BBP	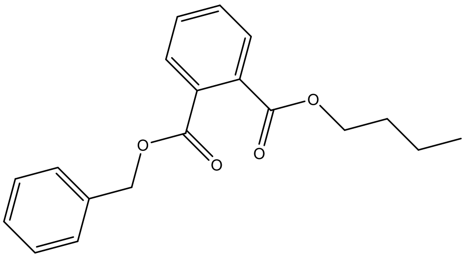
25	7911P	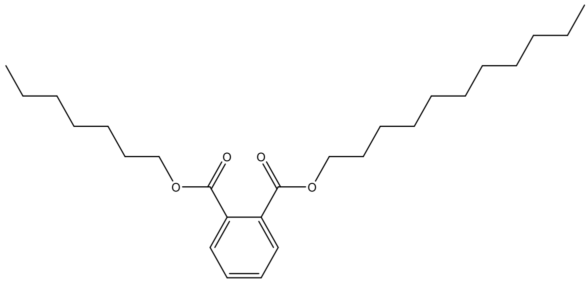

26	79P	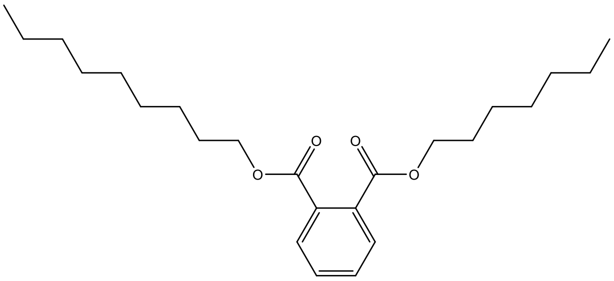
27	DNNP	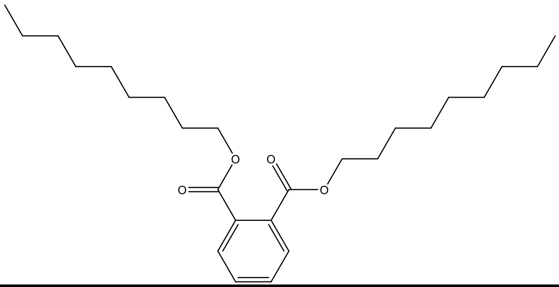

Table S2. Grid-SVR results under different numbers of descriptors.

n-feature	EEig07r	MWC10	PJI2	E3e	GATS7e	QYYv	IC2	EEig03r	ESpm04d	R5p	PJI3	Mor30m	tr_r2	cv_r2	te_r2
3	√	√	√										0.9576	0.9215	0.7583
4	√	√	√	√									0.9808	0.8853	0.7315
5	√	√	√	√	√								0.9363	0.8785	0.7616
6	√	√	√	√	√	√							0.9551	0.8938	0.7687
7	√	√	√	√	√	√	√						0.9526	0.8827	0.7246
8	√	√	√	√	√	√	√	√					0.9884	0.9169	0.8864
9	√	√	√	√	√	√	√	√	√				0.996	0.9375	0.8768
10	√	√	√	√	√	√	√	√	√	√			0.999	0.9531	0.8597
11	√	√	√	√	√	√	√	√	√	√	√		0.9997	0.9459	0.8595
12	√	√	√	√	√	√	√	√	√	√	√	√	0.9997	0.9217	0.8375

Table S3. Grid-RFR results under different numbers of descriptors.

n-feature	EEig07r	ESpm04d	E3e	QYYv	R7m+	MWC10	GATS7e	E2e	ALOGPS_logS	R5p	PJI2	EEig03r	tr_r2	cv_r2	te_r2
3	√	√	√										0.9646	0.7068	0.7364
4	√	√	√	√									0.9673	0.7119	0.6856
5	√	√	√	√	√								0.9589	0.6887	0.704
6	√	√	√	√	√	√							0.9613	0.7222	0.728
7	√	√	√	√	√	√	√						0.9623	0.7349	0.8071
8	√	√	√	√	√	√	√	√					0.9668	0.7305	0.7912
9	√	√	√	√	√	√	√	√	√				0.9584	0.6657	0.7008
10	√	√	√	√	√	√	√	√	√	√			0.9653	0.679	0.7048
11	√	√	√	√	√	√	√	√	√	√	√		0.9607	0.7522	0.6674
12	√	√	√	√	√	√	√	√	√	√	√	√	0.9647	0.723	0.6585

Table S4. GA-PLS Top 12 when molecular descriptors are screened to 25.

n-feature	EEig07x	Mor23v	R6m	Ve	ESpm06d	MATS3v	RDF075u	TIE	Mor08p	EEig07d	HATS2u	MATS8e	tr_r2	cv_r2	te_r2
4	√	√	√	√									0.8288	0.7493	0.8029
5	√	√	√	√	√								0.8274	0.7222	0.7995
6	√	√	√	√	√	√							0.8895	0.8267	0.8235
7	√	√	√	√	√	√	√						0.8834	0.7821	0.8101
8	√	√	√	√	√	√	√	√					0.8894	0.7853	0.8258
9	√	√	√	√	√	√	√	√	√				0.8754	0.826	0.8453
10	√	√	√	√	√	√	√	√	√	√			0.8855	0.8257	0.8212
11	√	√	√	√	√	√	√	√	√	√	√		0.8835	0.8436	0.805
12	√	√	√	√	√	√	√	√	√	√	√	√	0.8892	0.8527	0.7942

Table S5. GA-PLS Top 12 when molecular descriptors are screened to 13.

n-feature	R6m	Mor23v	MATS3v	EEig07x	Mor08p	HATS2u	TIE	EEig07d	Ve	ESpm06d	RDF075u	R3p	tr_r2	cv_r2	te_r2
4	√	√	√	√									0.8472	0.7623	0.7647
5	√	√	√	√	√								0.8279	0.5983	0.6384
6	√	√	√	√	√	√							0.8775	0.677	0.6567
7	√	√	√	√	√	√	√						0.869	0.7378	0.8463
8	√	√	√	√	√	√	√	√					0.8757	0.7853	0.7945
9	√	√	√	√	√	√	√	√	√				0.8836	0.8362	0.8187
10	√	√	√	√	√	√	√	√	√	√			0.8834	0.8339	0.8162
11	√	√	√	√	√	√	√	√	√	√	√		0.8835	0.8436	0.805
12	√	√	√	√	√	√	√	√	√	√	√	√	0.8722	0.8294	0.7687

Table S6. GA-SVR results under different numbers of descriptors.

n-feature	tr_r2	cv_r2	te_r2
3	0.8926	0.8527	-3.6424
4	0.9401	0.8881	-5.2259
5	0.9456	0.8915	-5.6212
6	0.9568	0.9074	-7.3445
7	0.9494	0.9081	-6.7311
8	0.9553	0.9004	-6.4896
9	0.9596	0.9016	-5.9159
10	0.9644	0.9136	-6.7167
11	0.9656	0.9122	-7.2258
12	0.9632	0.8936	-6.1998

Table S7. GA-RFR results under different numbers of descriptors.

n-feature	tr_r2	cv_r2	te_r2
3	0.9762	0.7901	0.2577
4	0.9761	0.8201	0.2664
5	0.9781	0.8113	0.0484
6	0.9697	0.8137	0.0438
7	0.9691	0.7887	0.0767
8	0.9707	0.7737	-0.0679
9	0.9731	0.7828	-0.0831
10	0.9636	0.7478	-0.0642
11	0.9695	0.7547	0.0276
12	0.9667	0.7633	0.0089

Table S8. R2 for different models on training set, cross-validation and test set.

Model Methodology			Based on 25 characteristics				Based on 14 characteristics			
			n-feature	tr_r2	cv_r2	te_r2	n-feature	tr_r2	cv_r2	te_r2
GA	PLS	GA-O	9	0.8754	0.826	0.8453	13	0.9599	0.7208	0.8507
		GA-RE	17	0.9933	0.9567	0.8805	11	0.9916	0.8869	0.9031
		GA-REC	20	0.9947	0.9032	0.8953	13	0.9768	0.9062	0.8138
	SVR	GA-O	18	1	0.6259	0.886	10	1	0.7082	0.8052
		GA-RE	9	1	0.8944	0.9834	8	1	0.8244	0.9651
		GA-REC	11	1	0.8649	0.8419	11	1	0.839	0.8769
	PCA+PLS	GA-O	3	0.8697	0.7294	0.7611	8	0.943	0.6796	0.7602
		GA-RE	10	0.9876	0.8871	0.8778	10	0.9792	0.8059	0.8621
		GA-REC	5	0.9497	0.8895	0.8126	11	0.9833	0.8359	0.7909
	PCA+SVR	GA-O	13	1	0.5727	0.8623	12	1	0.6409	0.8687
		GA-RE	6	1	0.8524	0.8926	9	1	0.8702	0.9539
		GA-REC	13	1	0.8018	0.7887	5	1	0.9182	0.9181

Table S9. SF projections for some potential plasticisers.

No.	Structure	SF Predicted Value
1	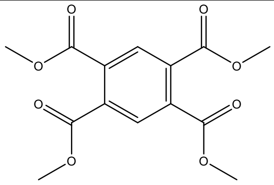	1.03
2	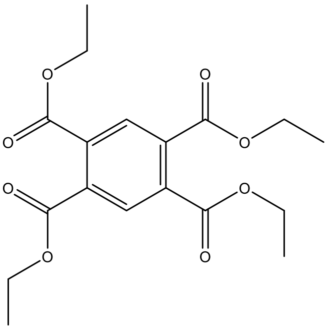	0.98
3	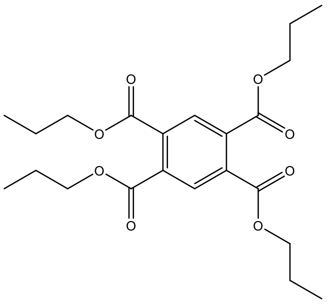	0.94
4	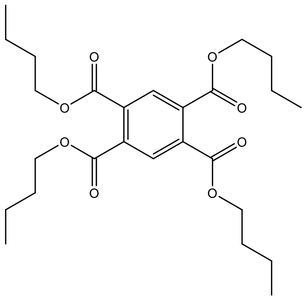	1.03
5	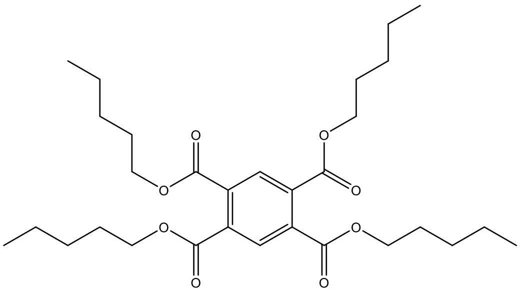	1.06
6	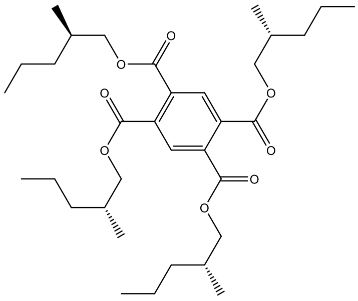	0.80

7	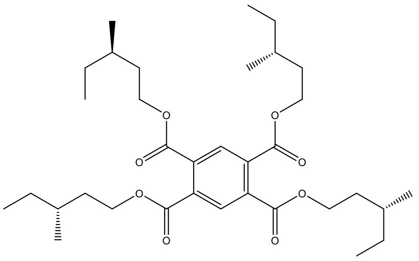	0.84
8	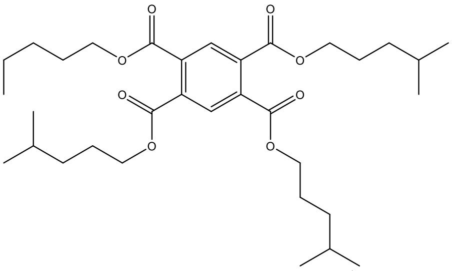	0.83
9	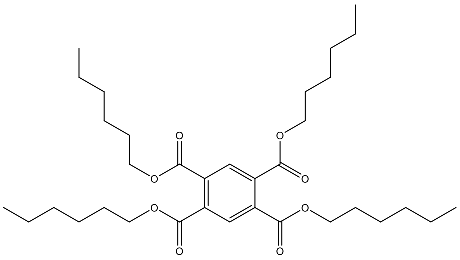	1.14
10	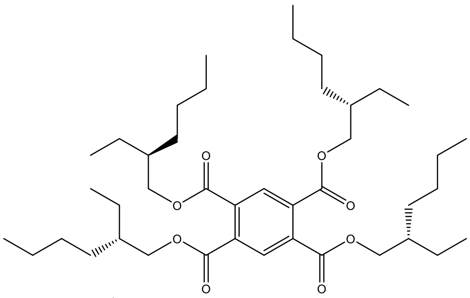	1.28
11	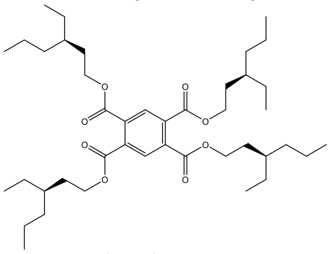	1.04
12	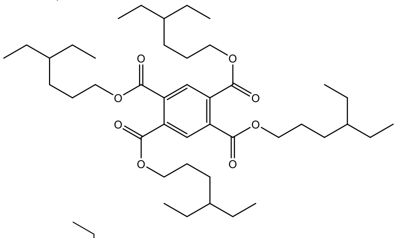	0.98
13	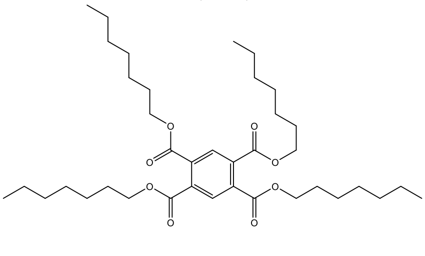	1.24

14	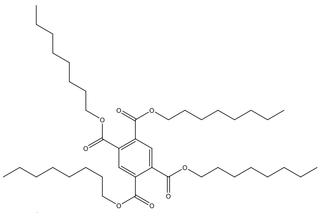	1.41
15	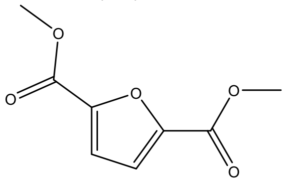	0.37
16	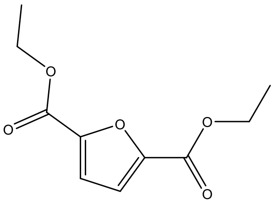	0.43
17	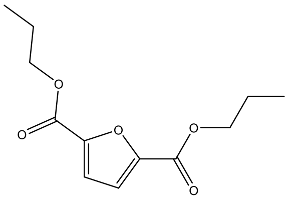	0.59
18	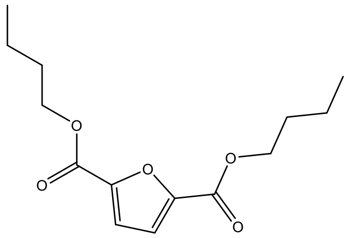	0.78
19	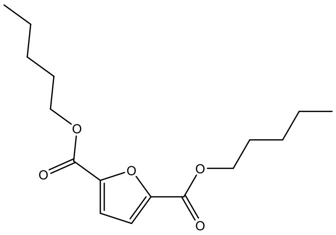	0.89
20	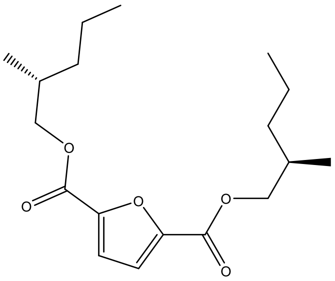	1.11
21	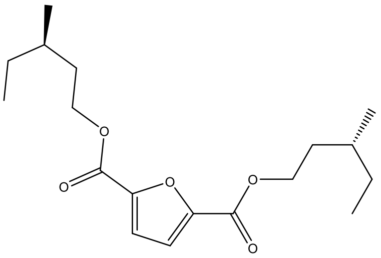	1.10

22	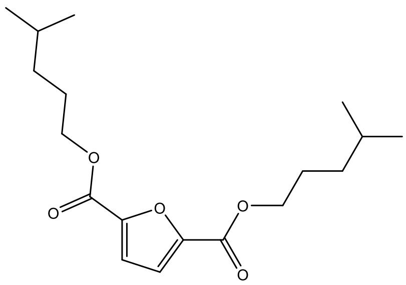	1.13
23	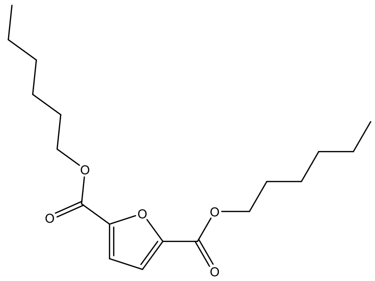	0.94
24	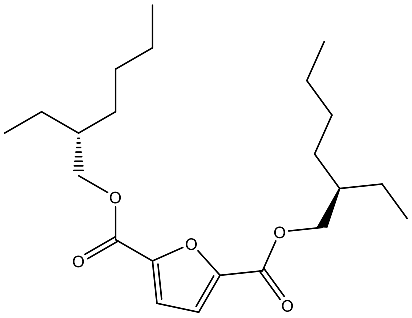	1.16
25	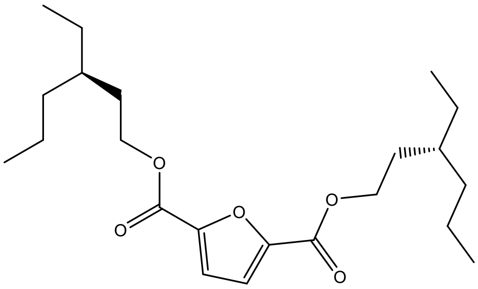	1.16
26	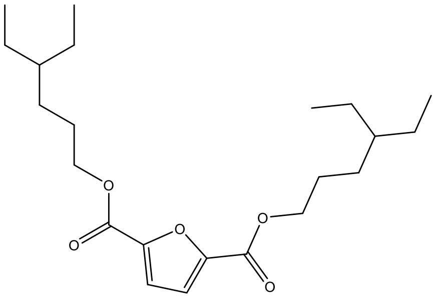	1.17
27	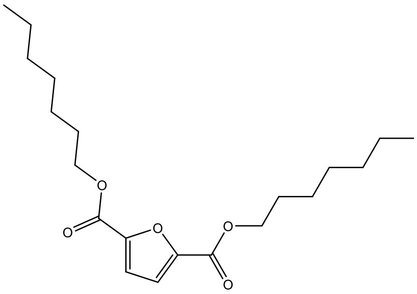	0.97

28	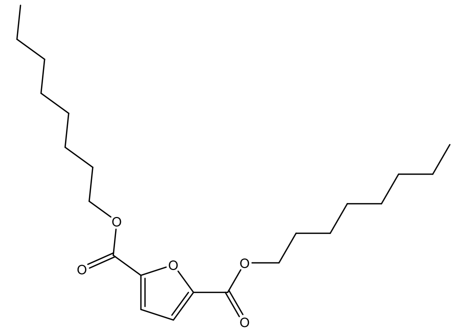	0.99
29	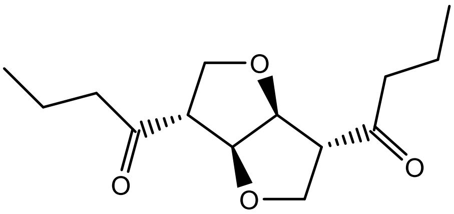	0.91
30	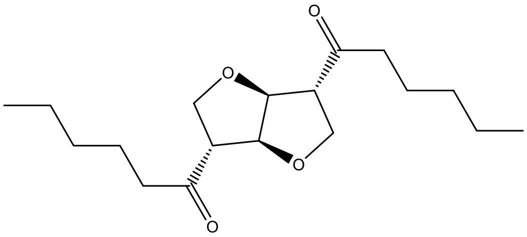	0.98
31	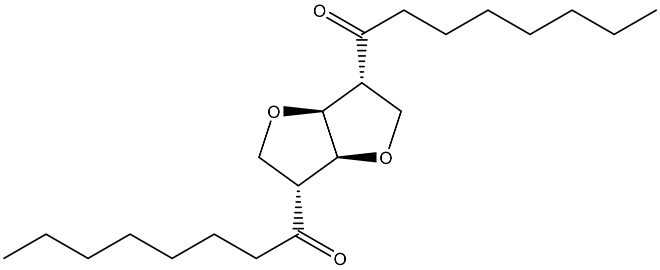	1.04
32	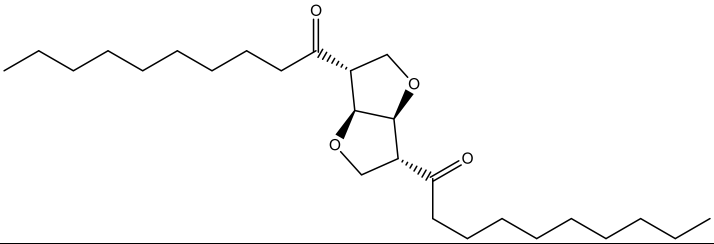	1.09
